# A Rare Case of Incidental Common Bile Duct Adenoma-Endoscopic Ultrasound Evaluation

**DOI:** 10.4274/balkanmedj.2017.1485

**Published:** 2018-07-24

**Authors:** Yana Valerieva, Ivan Lutakov, Branimir Golemanov, Georgi Jelev, Borislav Vladimirov

**Affiliations:** 1 Clinical Center of Gastroenterology, University Hospital “Queen Joanna-ISUL” Sofia, Medical University Sofia, Bulgaria; 2 Clinical Center of Gastroenterology, Department of Surgery, University Hospital “Queen Joanna-ISUL” Sofia, Medical University Sofia, Bulgaria

An 81-year-old female patient with rectal bleeding caused due to a 2-cm stalked sigmoid polyp was referred to our center for polypectomy. She had a history of chronic cholecystitis with several episodes of biliary colic without jaundice or fever since the past few years (she refused surgical treatment). Physical examination was insignificant, whereas laboratory tests revealed cholestasis. Abdominal ultrasound (US) revealed dilated intra- and extra-hepatic bile ducts–a common bile duct measuring up to 20 mm with an inhomogeneous mass within it with discrete acoustic shadowing; the gallbladder was enlarged and thick-walled, with microlithiasis. The patient was scheduled for sigmoid polypectomy and endoscopic retrograde cholangiopancreatography. The stalked sigmoid polyp was removed using an endoloop and a snare without any complications. The histology revealed tubulovillous adenoma with high-grade dysplasia. The endoscopic retrograde cholangiopancreatography showed a flat and sclerotic papillary orifice, with an irregular filling defect in the dilated common bile duct. A sphincterotomy was performed, and a part of a reddish, lobulated polyp (10 mm) with insignificant hemobilia was extracted using Dormia basket (Figure 1). The procedure was terminated to further evaluate the type and the extent of the lesion. The histology revealed an adenoma with low-grade dysplasia (Figure 2). Endoscopic ultrasound examination of the biliary tree (transduodenal view, linear probe GF-UCT180, Olympus; Aloka Alpha 7, Hitachi) showed a persisting dilation of the common bile duct with a hypoechoic, homogeneic, lobulated lesion measuring >45 mm “floating” in the bile. The tumor engaged about two-thirds of the common bile duct lumen, had broad vascularization, and was connected to the bile duct wall on a small surface, resembling a stalk (Figure 3a, 3b). No infiltration or significant lymph nodes were detected, and the patient was indicated for surgery. Through laparoscopic approach, a broad choledochotomy was performed revealing a large villous-looking polyp attached to the posterior–medial wall of the bile duct in the area of the cystic duct, followed by instrumental revision of the biliary tree. The polyp was removed through the site of choledochotomy, and the stalk area was coagulated, followed by cholecystectomy (Figure 4). In the 8 months of follow-up, the patient is symptom-free, with normal-sized bile ducts and normal laboratory tests.

An informed consent was signed by the patient for all medical procedures undertaken and publication purposes.

Benign lesions of the extra-hepatic biliary tree are very rare compared with malignancies and account for only 0.1% of biliary tract operations and 6% of all extra-hepatic bile duct masses (1). These adenomas are rare findings that are usually detected incidentally in removed gallbladders (cholelithiasis, chronic cholecystitis). However, they can occur anywhere in the extra-hepatic bile ducts with uncertain malignant potential, and there are still no guidelines for their management. A review conducted by Loh et al. (2) found that a total of 39 cases of bile tree adenomas have been reported till date. Different imaging modalities have been used for identifying these tumors with varying success rates, including contrast-enhanced ultrasound, intraductal ultrasound, and even ^18^F-fluorodeoxyglucose uptake (3,4). Endoscopic therapy has rarely been reported and is considered to be relevant only in patients with high risk of surgery (5).

Extra-hepatic bile duct adenoma is a rare entity, but it should be considered in the differential diagnosis of patients with a filling defect or obstruction of bile ducts. Endoscopic ultrasound is a safe and useful diagnostic modality in these cases.

## Figures and Tables

**Figure 1:**
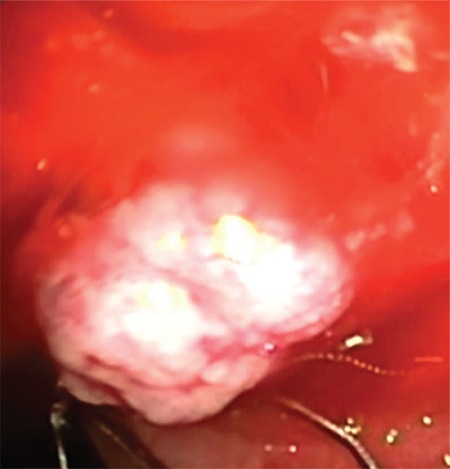
A piece of common bile duct adenoma being extracted using Dormia basket after papillotomy and endoscopic retrograde cholangiopancreatography.

**Figure 2:**
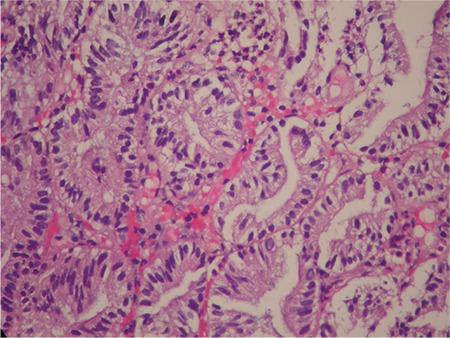
Common bile duct adenoma with low-grade dysplasia (H&E x40).

**Figure 3:**
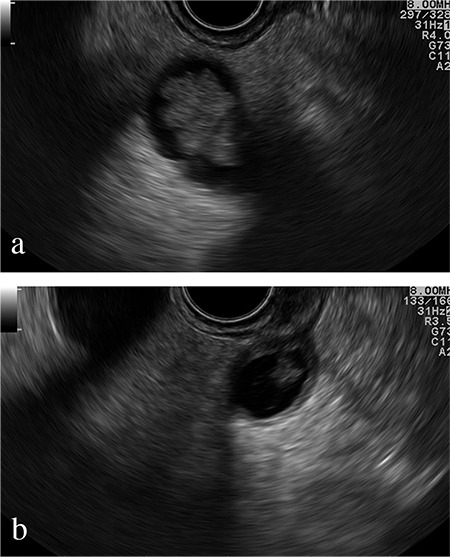
Transduodenal endoscopic ultrasound view of dilated common bile duct: lobulated soft tissue mass, “floating” in bile. Stalk is visible.

**Figure 4:**
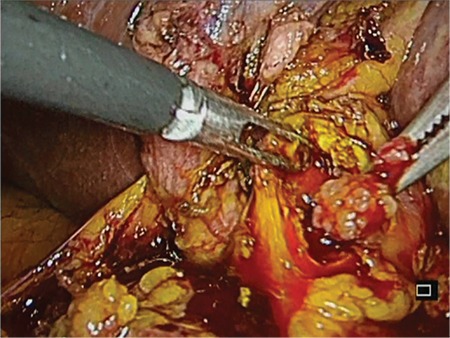
Laparoscopic broad choledochotomy: a villous-looking polyp attached to the posterior–medial wall of the bile duct.

## References

[1] Xu HX, Chen LD (2008). Villous adenoma of extrahepatic bile duct: contrast enhanced sonography findings. J Clin Ultrasound.

[2] Loh KP, Nautsch D, Mueller J, Desilets D, Mehendiratta V (2016). Adenomas involving the extrahepatic biliary tree are rare but have an aggressive clinical course. Endosc Int Open.

[3] Tefas C, Tanțău M, Szenftleben A, Chiorean L, Badea R (2015). Villous adenoma of the common hepatic duct: the importance of contrast-enhanced ultrasound and endoscopic retrograde cholangiopancreatography for relevant diagnosis. A case report and review of the literature. Med Ultrason.

[4] Hokonohara K, Noda T, Hatano H, Takata A, Hirota M, Oshima K, et al (2016). Tubular adenoma of the common bile duct with uptake in 18F FDG PET: A case report. Mol Clin Oncol.

[5] Munshi AG, Hassan MA (2010). Common bile duct adenoma: Case report and brief review of literature. Surg Laparosc Endosc Percutan Tech.

